# Fibronectin coating of oxygenator membranes enhances endothelial cell attachment

**DOI:** 10.1186/1475-925X-12-7

**Published:** 2013-01-28

**Authors:** Christian G Cornelissen, Maren Dietrich, Kai Gromann, Julia Frese, Stefan Krueger, Jörg S Sachweh, Stefan Jockenhoevel

**Affiliations:** 1 Department for Tissue Engineering & Textile Implants, Institute of Applied Medical Engineering, Helmholtz Institute of the RWTH Aachen University Hospital, Pauwelsstraße 20, 52074, Aachen, Germany; 2 Department of internal medicine – section for pneumology, RWTH Aachen University Hospital, Pauwelsstr. 30, 52074, Aachen, Germany

## Abstract

**Background:**

Extracorporeal membrane oxygenation (ECMO) can replace the lungs’ gas exchange capacity in refractory lung failure. However, its limited hemocompatibility, the activation of the coagulation and complement system as well as plasma leakage and protein deposition hamper mid- to long-term use and have constrained the development of an implantable lung assist device. In a tissue engineering approach, lining the blood contact surfaces of the ECMO device with endothelial cells might overcome these limitations. As a first step towards this aim, we hypothesized that coating the oxygenator’s gas exchange membrane with proteins might positively influence the attachment and proliferation of arterial endothelial cells.

**Methods:**

Sheets of polypropylene (PP), polyoxymethylpentene (TPX) and polydimethylsiloxane (PDMS), typical material used for oxygenator gas exchange membranes, were coated with collagen, fibrinogen, gelatin or fibronectin. Tissue culture treated well plates served as controls. Endothelial cell attachment and proliferation were analyzed for a period of 4 days by microscopic examination and computer assisted cell counting.

**Results:**

Endothelial cell seeding efficiency is within range of tissue culture treated controls for fibronectin treated surfaces only. Uncoated membranes as well as all other coatings lead to lower cell attachment. A confluent endothelial cell layer develops on fibronectin coated PDMS and the control surface only.

**Conclusions:**

Fibronectin increases endothelial cells’ seeding efficiency on different oxygenator membrane material. PDMS coated with fibronectin shows sustained cell attachment for a period of four days in static culture conditions.

## Background

In the European Union, 700 lung transplantations are performed for the treatment of lung failure per year [1]. Because organ shortage limits this number, 20% of patients on the waiting list for a transplant die every year [2]. Lung transplantation’s associated risks such as acute and chronic graft rejection, the need for life-long immunosuppression and a five year survival rate of just above 50% [2] lead to its classification as a treatment of last resort. Leading to transplantation early in life, Mucoviscidosis is the most frequent inborn cause of terminal chronic respiratory insufficiency with an incidence of 1 in 3000 live-births [3]. End stage treatment of other chronic respiratory diseases such as chronic obstructive pulmonary disease (COPD), idiopathic pulmonary fibrosis or pulmonary arterial hypertension also involves lung transplantation as the final treatment option.

Extracorporeal membrane oxygenation (ECMO) can replace the lungs’ gas exchange capacity until recovery or may be used as bridge-to-transplant in terminal lung failure [4]. However, limited hemocompatibilty associated with possible subsequent plasma leakage and loss of gas transfer capacity due to unspecific protein adsorption on the gas exchange membranes as well as inflammatory processes initiated by the foreign material’s surface limit its use for long-term application. Also, the limitations delineated above constrained the development of a fully implantable lung assist device.

Tissue engineering’s new strategies for enhancing biocompatibility comprehend methods that allow the fabrication of a confluent endothelial cell layer on biological and artificial surfaces [5]. The confluent endothelial layer of blood vessels regulates coagulation by tightly controlling the plasmatic coagulation cascade and the adherence of thrombocytes. The intact endothelium also orchestrates the delicate balance between pro- and anti-inflammatory stimuli at its surface, the complement cascade and leukocyte mediated processes being the most important ones. Hence, a confluent endothelium on the surface of a gas exchange membrane should be able to control the activation of coagulation and inflammation observed on current oxygenator membranes. In a tissue-engineering approach, this endothelialisation could be performed *in vitro* prior to the possible implantation of the device.

Material characteristics have hampered surface endothelialization in several instances. Bengtsson et al. implanted endothelialized mechanical heart valves made from pyrolytic carbon in pigs in the pulmonary position [6]. The endothelial cells were swept away by the blood flow within one hour. Herring et al. observed similar results for the endothelialization of vessel grafts fabricated from Polytetrafluoroethylene [7]. Still, extensive surface modification as proposed by Haverich et al. resulted in a confluent endothelial cell layer on Polymethylpentene (TPX) [8], a material employed in the production of gas exchange membranes. These membranes, also manufactured from Polypropylene (PP) and Polydimethylsiloxane (PDMS), have been developed with a focus on lowering adhesion of proteins and cells and endothelial cell seeding on these surfaces might not be straightforward. Regarding simpler adsorption strategies for protein coating onto oxygenator membranes and their effects on endothelial cell adhesion, growth and differentiation, no workup has been performed thus far that provides a head-to-head comparison of a wider array of adsorbed proteins on all currently used oxygenator materials.

Material characteristics and different protein coatings might influence endothelial cell adhesion and proliferation. Thus, membranes from TPX, PP and PDMS were coated with gelatin, collagen IV, fibroectin or fibrinogen. Initial cell attachment as well as cell proliferation were measured.

## Methods

### Membranes & protein coating

Polymethylpentene (TPX) and PP membranes were purchased from Goodfellow (Germany), cut into discs (diameter 15 mm) and fixed at the bottom of a 24-well microtiter plate (Becton & Dickinson, Germany). PDMS membranes were directly cast to the bottom of a microtiter plate using a two-part PDMS rubber (ELASTOSIL, Wacker Chemie, Germany). One week prior to protein coating, the membranes were sterilized using low-temperature hydrogen peroxide gas plasma (STERRADs 100S sterilisation system; Ethicon, Germany). Lyophilized proteins (all from Sigma, Germany) were dissolved in double-distilled water according to the manufacturer’s instructions at a concentration of 10 μg / mL except for gelatin, as it is standard to use it at a concentration of 20 μg / ml for cell culture. Following sterile filtration, 500 μl of the respective protein solution were used to coat the membranes for 24 hours at 37°C. Gelatin coating was performed for 15 minutes to avoid crystallization. Three samples of each possible membrane – protein combination were fabricated.

### Cell isolation and culture

Sheep carotid arterial endothelial cells were used for this experiment as sheep are a widely accepted animal model in cardiovascular tissue engineering. For cell isolation, carotid arteries were harvested from juvenile sheep deceased during other experiments under sterile conditions and immediately placed in sterile transport buffer (100 mM HEPES, 140 mM NaCl, 2.5 mM KCl, 10 mM glucose, 1% antibiotic/antimycotic solution (penicillin G-streptomycin-amphotericin B; Gibco, Karlsruhe, Germany); pH 7.4). The procedures used conform to the “guide for the care and use of laboratory animals” published by the US National Institutes of Health (NIH Publication No. 85-23, revised 1996).

Endothelial cells were isolated from the explanted carotid arteries. The artery was washed with phosphate-buffered saline (PBS; Gibco, Karlsruhe, Germany) before removing endothelial cells using 1 mg / ml collagenase (Sigma, Germany). Endothelial cells were re-seeded on 2% gelatin pre-coated tissue culture flasks in endothelial basal medium (PAA Laboratories GmbH, Pasching, Austria) supplemented with endothelial medium supplement (PAA Laboratories GmbH) and 1% antibiotic-antimycotic solution (penicillin-streptomycin-amphotericin B; Gibco) and maintained in a humidified incubator at 37°C and 5% CO_2_. Upon 80% confluence, cells were serially passaged using 0.25% trypsin/0.02% EDTA solution (Gibco). Cells in passage 4 were used for this study.

### Cell seeding

Cells were suspended in endothelial basal medium with supplement and seeded onto the protein coated membranes at a concentration of 5 * 10^4^ / cm^2^. Uncoated, tissue-culture treated wells on the same multiwell-plates served as positive controls while uncoated membranes served as negative controls. Cells were maintained in a humidified incubator at 37°C and 5% CO_2_. Medium was changed on day 2.

### Conventional light microscopy

Untreated samples were analyzed with routine bright field microscopy (AxioImager; Carl Zeiss GmbH, Germany) and scaled images were acquired using a digital camera (AxioCam MRm; Carl Zeiss GmbH). Software assisted cell counting was performed on the acquired images (Image-Pro Plus Version 6.2; Media Cybernetics Inc., MD, USA).

### Statistics

Continuous variables are expressed as mean ± SD or median and interquartile range in parenthesis unless stated otherwise. Due to the small sample size the analysis is rather descriptive. Data analysis was performed using commercially available software (SAS enterprise guide version 4, SAS Institute Inc., NC, USA & Microsoft Excel 2007, The Microsoft Corporation, USA).

## Results

**Figure 1:**
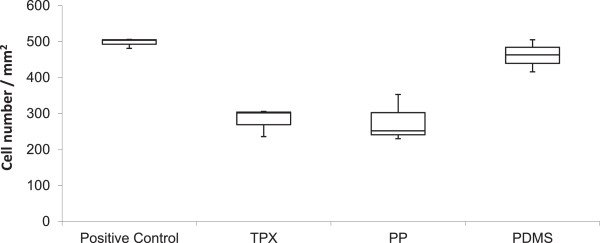
**Box plot of cell density after 24 hours of culture on fibronectin coated materials.** Median – bar, Quartiles – boundaries of box, maximum and minimum – whiskers, intial seeding 500 cells / mm^2^. This diagram illustrates the high seeding efficiency on fibronectin coated PDMS which is on par with seeding efficiency on standard tissue-culture treated labware (control). Seeding on fibronectin coated PP and TPX is less efficient.

Endothelial cell seeding efficiency is within range of tissue culture treated controls for fibronectin treated surfaces only (Table 1). After 24 hours 72% (± 15%) cells seeded on control surfaces adhere while 93% (± 5%) adhere on fibronectin coated PDMS (Figure 1). Adherence of cells on fibronectin coated PP and TPX is 55% (± 7%) and 56% (± 4%), respectively. All other surface coating / material combinations lead to attachment rated below 25%. Uncoated membranes allow for the attachment of less than 20% of the seeded cells.

**Table 1:** **Median and interquartile range of cell numbers / mm**^**2 **^**with respect to material, protein coating and culture time**

**Material**	**Protein**	**Day 1 (cells / mm**^**2**^**)**	**Day 2 (cells / mm**^**2**^**)**	**Day 4 (cells / mm**^**2**^**)**
Control		504 [493 – 505]	559 [543 – 589]	673 [626 – 690]
Polypropylene (PP)	No Coating	99 [95 – 104]	93 [82 – 93]	130 [114 – 136]
	Gelatin	75 [72 – 82]	127 [105 – 133]	343 [278 – 348]
	Fibronectin	252 [241 – 303]	215 [188 – 299]	439 [360 – 466]
	Fibrinogen	109 [100 – 110]	105 [99 – 123]	194 [169 – 209]
	Collagen IV	98 [89 – 103]	108 [99 – 116]	182 [173 – 288]
Polymethyl-pentene (TPX)	No Coating	57 [49 – 59]	15 [11 – 21]	35 [23 – 39]
	Gelatin	28 [27 – 29]	30 [22 – 31]	3 [3 – 5]
	Fibronectin	302 [269 – 304]	230 [211 – 246]	114 [110 – 115]
	Fibrinogen	50 [41 – 55]	25 [25 – 26]	41 [37 – 44]
	Collagen IV	43 [39 – 46]	41 [27 – 42]	21 [21 – 23]
Polydimethyl-Siloxane (PDMS)	No Coating	2 [2 – 6]	1 [1 – 4]	1 [1 – 1]
	Gelatin	9 [6 – 13]	4 [4 – 65]	2 [2 – 74]
	Fibronectin	463 [440 – 484]	572 [531 – 588]	622 [611 – 623]
	Fibrinogen	10 [9 – 11]	4 [4 – 5]	2 [2 – 3]
	Collagen IV	8 [8 – 9]	6 [5 – 8]	2 [2 – 5]

Footnote: The typical maximum of cell density of endothelial cells *in vivo* and under *in vitro* culture conditions is 1,000 cells / mm^2^.

**Figure 2:**
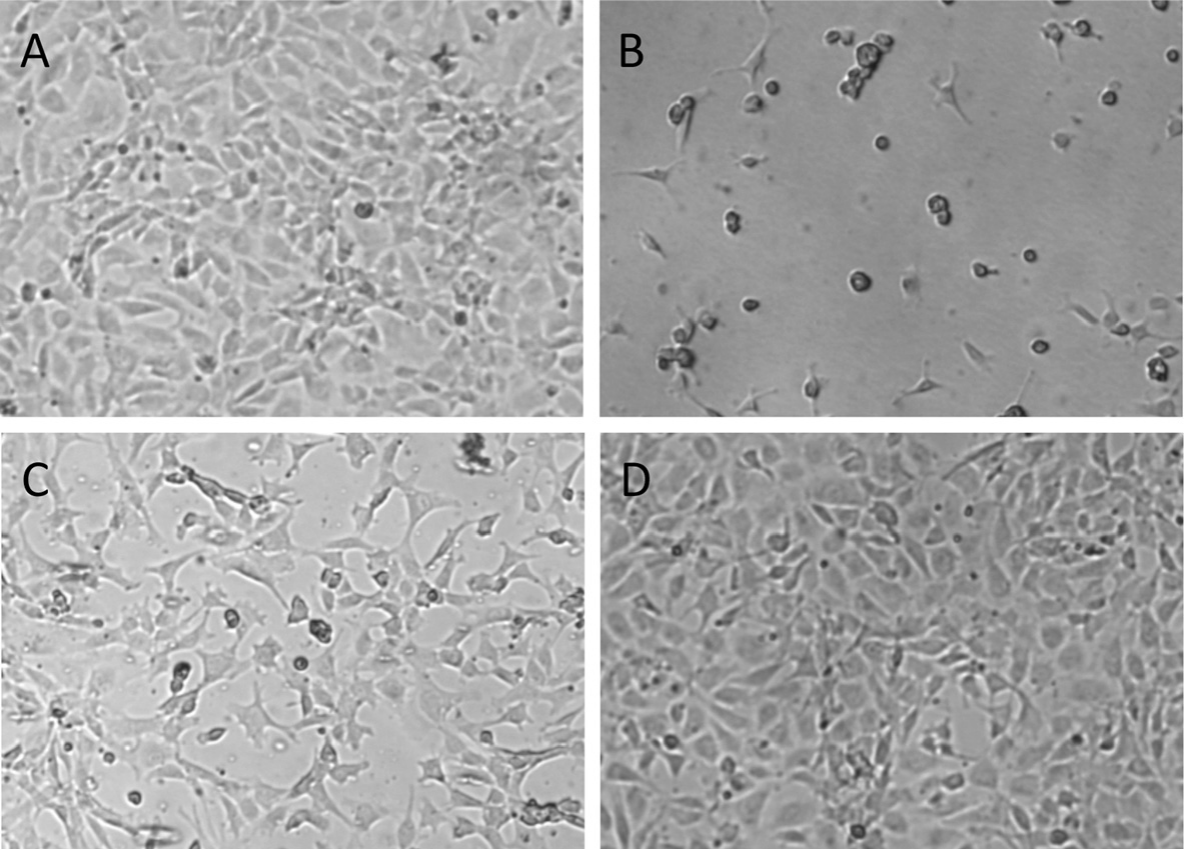
**Phase contrast microscopy of cultured endothelial cells on day 4. A** – tissue culture plastic, **B** – Polymethylpentene, **C** – Polypropylene, **D** – Polydimethylsiloxane. Scale 100 μm. All materials except A are coated with fibronectin. Endothelial cells form confluent monolayers after 4 days of culture on tissue culture plastic (**A**) and PDMS (**D**) but neither on TPX (**B**) nor on PP (**C**).

**Figure 3:**
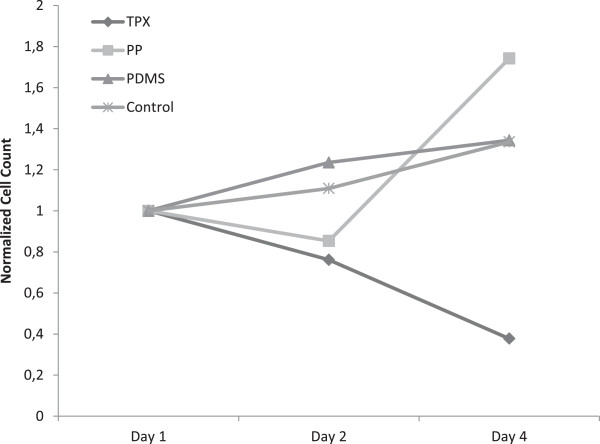
**Normalized cell count on fibronectin coated materials.** Cell numbers were normalized to the number of cells after 24 hours of culture to illustrate changes in cell number. A steady but slow increase in cell number is observed for cells on tissue-culture treated labware as well as fibronectin coated PDMS. On TPX, the cell number continuously drops while on PP, the low initial cell attachement leads to a marked increase in cell number.

Cells seeded onto the control surface proliferate throughout the culture period with cell numbers increasing by 60% (± 30%) during four days. On day four, cells are almost confluent on the control surface (Figure 2). Cells also grow to confluence on fibronectin coated PDMS while cell proliferation remains lower at 34% (± 2%) due to the high initial cell density (Figure 3). Cell morphology reveals the “cobblestone pattern” on both surfaces, which is typical of differentiated endothelial cells in monolayer culture (Figure 2).

Cells seeded onto fibronectin coated PP also proliferate well with an increase in cell number of 50% (± 22%) from day 2 to day 4 (Figure 3). Keeping in mind the lower seeding efficiency, this does not result in a confluent endothelial cell layer (Figure 2). Cell morphology does not show the typical “cobblestone pattern”. Instead, elongated, branching cells make dedifferentiation of the endothelial cells obvious.

Cells seeded on fibronectin coated TPX begin to detach from the surface from day 1 to day 2 already. Only 38% (± 6%) of the initially seeded cells remain on the surface after 4 days (Figure 3). Cells show signs of dedifferentiation after the first day of culture already (Figure 2).

Cells seeded onto uncoated membranes or membranes coated with gelatin, fibrinogen or collagen IV all reveal a dedifferentiated morphology with elongated, branching cells. On uncoated as well as gelatin coated PP, cells are able to proliferate with an increase in cell number of 30% (± 12%) and 457% (± 15%) respectively. Still, no confluent cell layer is present and cells exhibit signs of dedifferentiation.

## Discussion

Among other design considerations, one important limiting factor in the development of an implantable lung assist device is the polymeric surface of commercially available oxygenators, causing limited hemocompatibility, activation of the coagulation and complement system, plasma leakage and protein deposition and, finally, loss of function. To overcome these issues and render these systems more suitable for long-term use, endothelialisation of these membranes may be a solution. In a tissue-engineering approach, this endothelialisation could be performed *in vitro* prior to the possible implantation of the device. While small defects in the oxygenator’s surface can lead to an increased activation of the blood clotting cascade in oxygenators currently used, a living cell lining might be able to repair endothelial defects.

Endothelial cell seeding depends on surface characteristics, as became obvious in the efforts of Bengtsson et. al and Herring et al. to endothelialize a mechanical heart valve and a synthetic vessel prosthesis [6,7]. In the present study, we demonstrate that seeding of endothelial cells to uncoated oxygenator membranes is ineffective. We also show that surface treatment by adsorption of gelatin, fibrinogen or collagen IV does not enhance the cell seeding process, but that adsorption of fibronectin to oxygenator membranes increases cell attachment. Fibronectin coated PDMS emerges as the surface best supporting endothelial cell attachment. A confluent endothelial cell layer develops on fibronectin coated PDMS and the control surface only. Similarly, cells display a “cobblestone pattern”, which is a sign of well differentiated endothelial cells, throughout the culture period only on fibronectin coated PDMS and the control surface.

Unlike these results, fibronectin [9], gelatin [10], fibrinogen [11] and collagen IV [12] are all in use for coating cell culture surfaces and generally allow endothelial cell attachment and growth. They do also support differentiation of immature cells towards endothelial lineage. Thus, the low attachment rates, the detachment and the dedifferentiated appearance of the seeded endothelial cells are likely due to low protein adsorption onto the materials’ surfaces. This is rather unsurprising, as oxygenator membranes have been designed and optimized for low protein adsorption to enhance hemocompatibility and to avoid a decrease in gas transfer due to protein deposition. However, fibronectin is able to adsorb to PP and PDMS well enough to support initial cell attachment. On PDMS, this adsorption is stable enough to support a confluent endothelial cell layer for 4 days.

Polydimethylsiloxane (PDMS) is in use for microfluidic applications and is known to well support extensive surface modification [13]. Proteins and peptides facilitating cell culture adhere to PDMS by adsorption or covalent bonds. Wang et al. immobilized fibronectin and collagen on PDMS by adsorption and showed good cell attachment and growth rates for intestinal epithelial cells [14] while Lee et al. demonstrated growth of human umbilical artery endothelial cells on PDMS coated with fibronectin by adsorption [15].

Plasma or aggressive chemicals create reactive groups on PDMS that are highly unstable due to its low glass transition temperature of -120°C [16], but allow for further covalent immobilization of proteins. Sui et al. demonstrated this approach by covalently immobilizing RGD peptides onto PDMS, which resulted in adequate attachment of A427 cells (a colon cancer cell line) [17]. Feinberg et al. employed this approach to immobilize fibronectin in a microprinted dot array onto PDMS that supported an endothelial layer of an exceptionally high density for a culture period of up to 18 days [18].

Singular reports exist that extensive surface modification allows for endothelial cell attachment on oxygenator membranes. Haverich et al. coated TPX membranes by adsorbing a combination of human albumin and sodium-heparin to the surface, while N-(3-dimethylaminopropyl)-N0-ethylcarbodiimide hydrochloride allowed for subsequent cross-linking of the coating. This resulted in a confluent endothelial cell layer on TPX [8]. This process is highly complex and creates a protein coating that is multilayered, which might well lead to a decrease in gas transfer, as is well known for protein deposition on oxygenator membranes [19].

Takagi et al. coated PP hollow fiber membranes with fibronectin by adsorption or covalent bonding, reporting confluent cell layers after 1 day of culture. Still, no data on long-term stability exceeding one day of culture is given [20].

Regarding simpler adsorption strategies for protein coating onto oxygenator membranes and their effects on endothelial cell adhesion, growth and differentiation, no workup has been performed thus far that provides a head-to-head comparison of a wider array of adsorbed proteins on all currently used oxygenator materials. The present study reveals that adsorption of gelatin, fibrinogen or collagen IV does not support a confluent endothelial cell growth on currently used oxygenator membrane materials. Adsorbed fibronectin does allow for initial cell attachment on all tested oxygenator membrane materials and endothelialization sustains for a period of 4 days on fibronectin adsorbed to PDMS, but not on any other tested membrane material.

## Conclusions

Our results pave way for further analysis of simple protein adsorption procedures to PDMS surfaces and underline the necessity of more complex coating strategies for other oxygenator membrane materials to allow endothelialization as a first step towards the development of an implantable lung assist device.

## Competing interests

The authors declare that they do not have any financial or non-financial competing interests.

## Authors’ contributions

CGC, MD and SJ conceived the concept and design of the study. Experiments were performed by MD, JF and KG. CGC and SK performed the data analysis. CGC, MD and JS wrote the manuscript. All authors read and approved the final manuscript.
